# Editorialets

**Published:** 1905-10

**Authors:** 


### Editorialets.

HOLLAND'S MAGAZINE, published at Dallas, Texas, is rapidly taking a high place in magazinedom as the most fitting Southern representative of magazine literature.

---

DR. JAMES T. JELKS, of Hot Springs, Ark., and Miss Belle McKaskle, of Pony, Mont., were married in Butte, Mont., September 7. They will be at home to their friends after October 1st, 480 Prospect avenue, Hot Springs, Ark.

---

DR. B. M. WORSHAM, superintendent State lunatic asylum, at Austin, Texas, has just recovered from an attack of appendicitis, and has gone to New York to have the thing cut out.

---

THE sixty-first quarterly meeting of the Austin District Medical Society (now the Seventh Councilor's), was held in Austin September 21, with a good attendance. Dr. F. Paschal, of San Antonio, read a paper on resection of knee joint, giving cases; Dr. W. Neal Watt, of Austin, a valuable paper on Tetanus; Dr. W. J.

Mathews, of Austin, one on Ice Pack in Pneumonia; Dr. M. M. Smith, of Austin, made a talk on Sanitarium Treatment of Tuberculosis, and Dr. S. E. Hudson, of Austin, late delegate from State Medical Association to the Portland meeting of A. M. A., made an interesting talk on what was done at that meeting. San Antonio being now outside of the district (No. 7), her physicians heretofore members, are cut off. Dr. Paschal was by unanimous vote made an honorary member. He will amplify his paper and read it at Fort Worth next April, at the meeting of the State Medical Association.

---

THE next meeting of the Northwestern Texas Medical Association will be held at Bowie, Texas, October 10th. A full attendance and a rousing meeting are expected. Those I. T. and North Texas fellows are loyal and enthusiastic. Can't be there myself; wish I could. Dr. A. R. Lewis, of Ryan, I. T., is the President.

---

MEETING POSTPONED.—Owing to the quarantine restrictions in a number of the States, the meeting of the Southwestern Tri-State Medical Association of Oklahoma, Indian Territory and Texas has been postponed from the 17th and 18th of October to the 8th and 9th of November, 1905. This meeting will be held in Oklahoma City and reduced rates of 50c plus one regular fare for the round trip has been obtained on all roads leading to Oklahoma City. Oklahoma City has first class hotel faciilties and a large attendance at this meeting is expected.

---

THE next regular meeting of the Board of Medical Examiners for the State of Texas will be held in San Antonio, Texas, on October 17, 18 and 19.

DR. T. T. JACKSON, Secretary.

San Antonio, Texas.

---

MARRIAGE.—Dr. Harry O. Sappington, of Galveston, Texas, and Miss Margaret G. Fay, a graduate of the University Hospital Training School for Nurses, were married in Altoona, Pa., on August 23d. Dr. and Mrs. Sappington will reside in Galveston.

---

THE seventh of Battle & Co.'s (St. Louis), series of twelve illustrations of the Intestinal Parasites, will be sent free, to physicians, on application.

THE twentieth semi-annual meeting of the Brazos Valley Medical Association will be held at Rockdale, Texas, on the second Tuesday and Wednesday in November, the 14th and 15th days. This meeting will be the most important ever held by the Association, and a full attendance is earnestly requested. All physicians cordially invited.

DR. H. T. COULTER, Rockdale,

President.

W. B. BRIGGS, Easterly,

Secretary and Treasurer.

---

DR. Q. C. SMITH, for over a quarter of a century a prominent practicing physician at Austin, Texas, has removed to San Diego, Cal., his former home long ago. His son, Dr. Stephen Smith is doing a fine dental practice there. My best wishes attend Q. C.

---

Fastidious was Dr. Carlisle,
He dressed in the nobbiest stysle;
If a lady he'd meet in crossing the street,
He'd dexterously doff his new tisle.

---

Busy was old Dr. Pugh,
He had more than one man could dugh,
But he went day and night for everything in sight,
And of funerals,—well, he had not a fugh.

---

Then there was that old fellow Talliafero (Toliver)
He hailed from up about Bolliafero;
He drove an odd pair, a mule and a mare,
And he had a young cub named Oliafero.

---

BE GOOD, DO IT NOW.—Every bill has been mailed to subscribers, and prompt responses are requested. It is sometimes inconvenient to get a postal money order, express order or bank exchange, and remittance is postponed to a more convenient season and forgotten. Doctor, just write out your personal check on your local bank while you have my bill in hand, pin them together and fire it in. "Be good, do it now."

---

THE cleaning of the Augean stables was not a circumstance compared to exterminating the mosquitoes in the thirty-mile wide swamps in the midst of which New Orleans is situated; a swamp that is always full of stagnant water and which, in many places, would bog a rabbit. It can not be drained. The hope is utopian. Kill every mosquito in New Orleans today and double as many would be there the next day. Optimism is a good quality tho'.

---

WANTED, TO TRADE, HOUSTON PROPERTY FOR COUNTRY PROPERTY.—Dr. Joseph Greer, of Humble, Texas, wants to trade a four-room cottage in Houston Heights, two lots, servant's house, barn, buggy house, and two nice, dry lots on Montgomery avenue, city of Houston, with a complete office outfit, carpets, matting, chairs, tables, static machine, X-ray outfit, Betz hot-air apparatus, massage outfit, and electric baths, for good property in a thrifty country and town. Will trade office outfit separate if desired. No incumbrance on any of the property and title perfect. Write to Dr. Joseph Greer, Humble, Texas.

---

QUARANTINE CHANGES.—Dr. B. U. Sims, quarantine inspector at El Paso, has resigned to go in the United States Army Medical Corps. He is succeeded by Dr. P. J. Shaver, the quarantine inspector at Sabine Pass, and Dr. W. R. P. Thompson of Smithville, who has been a floating inspector in the quarantine service, takes charge of the Sabine Pass station. Dr. J. F. Eaves, quarantine inspector at Rockport, is on detached service at Marshall, Texas, and Dr. Hicks Florence, who has been on detached service, has returned to his station at Brownsville.

---

DR. MIMS, chemist of the New Orleans Board of Health, has promulgated what he calls a "Culicide" (Culicicide or Culicifuge?) composed of equal parts of gum camphor and chrystalized carbolic acid. The Board is experimenting with it.

